# Quantitative EEG findings in patients with acute, brief depression combined with other fluctuating psychiatric symptoms: a controlled study from an acute psychiatric department

**DOI:** 10.1186/1471-244X-8-89

**Published:** 2008-11-11

**Authors:** Marte Helene Bjørk, Trond Sand, Geir Bråthen, Olav M Linaker, Gunnar Morken, Brigt M Nilsen, Arne Einar Vaaler

**Affiliations:** 1 Department of Neurology and Clinical Neurophysiology, St Olavs Hospital, Trondheim, Norway; 2 Department of Neuroscience, Faculty of Medicine, Norwegian University of Science and Technology, Trondheim, Norway; 3 Division of Psychiatry, Department of Research and Development, St Olavs Hospital, Trondheim, Norway; 4 Østmarka Psychiatric Department, St Olavs Hospital, Trondheim, Norway

## Abstract

**Background:**

Patients with brief depressive episodes and concurrent rapidly fluctuating psychiatric symptoms do not fit current diagnostic criteria and they can be difficult to diagnose and treat in an acute psychiatric setting. We wanted to study whether these patients had signs of more epileptic or organic brain dysfunction than patients with depression without additional symptomatology.

**Methods:**

Sixteen acutely admitted patients diagnosed with a brief depressive episode as well as another concurrent psychiatric diagnosis were included. Sixteen patients with major depression served as controls. Three electroencephalographic studies (EEG) were visually interpreted and the background activity was also analysed with quantitative electroencephalography (QEEG).

**Results:**

The group with brief depression and concurrent symptoms had multiple abnormal features in their standard EEG compared to patients with major depression, but they did not show significantly more epileptiform activity. They also had significantly higher temporal QEEG delta amplitude and interhemispheric temporal delta asymmetry.

**Conclusion:**

Organic brain dysfunction may be involved in the pathogenesis of patients with brief depressive episodes mixed with rapidly fluctuating psychiatric symptoms. This subgroup of depressed patients should be investigated further in order to clarify the pathophysiology and to establish the optimal evaluation scheme and treatment in an acute psychiatric setting.

## Background

The psychiatric acute and intensive care units serve a broad spectrum of acutely admitted patients. A limited number of patients presents with brief depressive periods intervened with rapidly alternating psychiatric symptoms more typical of other psychiatric diagnoses than depression. These patients fail to meet current diagnostic criteria for affective disorders and the optimal treatment is not established. For the purpose of the present study we call this condition Acute Unstable Depressive Syndrome (AUDS) and the specific criteria are defined in the methods section.

It is important to study subsyndromes of depression with EEG and other imaging techniques as possible differential diagnoses in such patients may be psychiatric disorders associated with epilepsy. Psychiatric disorders associated with epilepsy seem to be clinically distinct [1], and affective disorders and depression, often with atypical clinical presentations failing to meet current DSM-IV criteria, are the most frequent [2,3]. Studies of epilepsy or other organic brain dysfunctions in psychiatric acute departments are sparse [4]. However, it is important to identify patients with atypical depressive syndromes because treatment with traditional antidepressants might induce cycle acceleration in some patients with affective disorders [5,6]. Recognition of organic brain syndromes, like epilepsy, is important in acute wards in order to identify patients who may benefit from treatment with antiepileptic drugs [7].

Electroencephalography (EEG) is the best method to diagnose epileptic seizures and syndromes. EEG may also be useful in treatment selection [8,9] and classification [10,11] of mood disorders. Subgroups with different familiar characteristics, clinical features, and prognosis have also been identified by EEG methods within a psychiatric population [12]. Reasonably accurate separations of patients with primary depression from healthy individuals with quantitative EEG (QEEG) multivariable neurometric methods has been repeatedly demonstrated and replicated in large samples by one research group [13].

We aimed to use prospective and blinded analysis of EEG and QEEG [14,15] to investigate whether the AUDS group had more epileptiform and slow (delta and theta) activity in the frontal, temporal and posterior brain regions than major depressive patients in the course of an acute admission. We also aimed to study if regional alpha activity, posterior alpha frequency and regional QEEG asymmetry differed between the two groups. By this study we hoped to clarifiy the pathophysiology and nosological status of these unstable depressive patients.

## Methods

### Population

The following criteria had to be fulfilled for inclusion in the study group (AUDS patients): A history of a rapidly developing psychiatric condition starting within the last 14 days. Within these 14 days the patient had at different times shown symptoms that met criteria for at least two diagnoses in the Diagnostic and Statistical Manual of Mental Disorders, fourth edition (DSM-IV) Axis 1 categories (with exception of the time criterion) [16]. One of these new DSM-IV Axis 1 categories had to be a depressive episode (with exception of the time criterion) defined as Montgomery and Aasberg Depression Rating Scale (MADRS) ≥ 20 [17]. Patients with one of the following conditions were excluded: a psychiatric condition due to direct effects of acute intoxication, dementia or mental disabilities to such a degree that informed consent could not be obtained, unstable personality disorder with identical symptoms at former admissions, or unable to speak English or Norwegian. From a total of 1984 admittances we evaluated 1038 persons for inclusion. Twenty-eight fulfilled criteria for inclusion in the study group. Twelve were not included (not willing to participate, informed consent not possible to obtain, mentally disabled to an extensive degree, or not speaking Norwegian or English). Sixteen patients entered the study group (AUDS). Mean age was 32.1 years (Table 1). Their co-diagnoses (with exception of the time criterion) were DSM-IV 298.8 "brief psychotic disorder" (nine patients), DSM-IV 300.1 "panic disorder" (four patients) and DSM-IV 296.0 "single manic episode" (three patients).

**Table 1:** Clinical background data

	AUDS patients	MDE patients	p-value
	n = 16	N = 16	

Age^a^	32.1 (11.4, 19–58)	32.8 (13.0, 18–62)	0.99^e^

Gender (men)	6 (38%)	6 (38%)	1.00^f^

Neuroleptic medication^g^	13 (81%)	14 (88%)	1.00^f^

Anti-epileptic medication	11 (62.5%)	1 (6.25%)	0.001^f^

Alcohol abuse^b^	4 (27%)^c^	4 (31%)^d^	1.00^f^

Drowsiness	11 (69%)	11 (69%)	1.00^f^

Number of subjects (%)

^a ^Mean (SD, range)

^b ^The Alcohol Use Disorders Identification Test (AUDIT) ≥ 8

^c^n = 15 ^d^n = 13

^e^Mann-Whitney test ^f^Fisher exact test

^g ^anti-depressive and anti-psychotic medication

AUDS: Acute Unstable Depressive Syndrome. MDE: Major Depressive Episode

The control group consisted of 16 acutely admitted sex- and age (+/- 5 years), matched patients meeting criteria (MADRS ≥ 20) for current Axis 1 major depressive episode (MDE). Mean age was 32.8 years (Table 1). The first patient meeting such criteria after inclusion of a study group patient was recruited to the control group. Written consent was obtained from all the patients prior to inclusion. The study was performed in accordance with the ethical standards laid down in the 1964 Declaration of Helsinki. The Regional Ethical Committee approved of the study.

### Other Assessments

We examined the patients on day 2, day 4–6, day 14–16 and after 3 months. Alcohol use was assessed with The Alcohol Use Disorders Identification Test (AUDIT) [18]. Illicit drug use was assessed with the Structural Clinical Interview for DSM-IV (SCID-1) [19], and urine samples were screened for drugs at admittance. Current medication concentrations were analyzed in blood samples at admittance.

### EEG recording and visual analysis

We obtained thirty minutes of eyes-closed EEG-videometry on day 2, day 4–5, and day 8–10 after admittance. Three tests were performed in order to increase the probability of detecting epileptiform activity as well as to evaluate time-related effects caused by e.g. drug use. They were all done at the same time of day (+/- 1.5 hours). We used digital equipment (Nervus 2.4 or 3.0) with 21 EEG electrodes placed according to the 10/20 international system, electrocardiography (ECG) and eye movement detection channels. Standard photic stimulation (1 to 60 Hz) and 3 min of hyperventilation were performed. Data loss occurred in one AUDS patient in test 2, and in 3 MDE patients in test 3. An experienced EEG physician blinded for the diagnosis interpreted and performed a clinical EEG analysis with classification of alpha frequency, epileptic spike activity and focal-and generalized slow activity. Eye opening and closing were performed every minute in order to control vigilance fluctuations. An experienced EEG technician noted presence of drowsiness in the recording. The technician, the physician and others doing the analyses and statistical work were blinded regarding the patient group.

### QEEG of analysis of baseline activity

We selected artefact free epochs of 4 seconds duration. Epochs with eyeblinks, muscle artefacts, electrode/movement artefacts, epileptiform activity and drowsiness were excluded. The total duration of such epochs was between 20 s and 60 s. We used the average reference montage, a band pass filter of 0.5 Hz – 70 Hz and a 50 Hz notch filter. Sixteen channels (F3, F4, F7, F8, T3, T4, T5, T6, O1, O2, C3, C4, P3, P4, T1 and T2) were exported to MATLAB™. The time/amplitude series had a sampling frequency of 256 Hz. For each selected EEG-segment we eliminated the start/end-discontinuities by using a cosine window. Fast Fourier Transform (FFT) was performed on four second samples (N = 1024 points), with two seconds overlap. This transform computes the power spectrum (EEG-power in μV^2 ^on the y-axis as a function of frequency in Hz in the x-axis). The average amplitude spectra (square root of power) across all the 4 seconds long samples were thereafter calculated and smoothed. Band amplitudes were calculated as the sum of spectral amplitudes for all bins in the frequency band and used in the statistical comparisons. The frequency bands were delta (1–3.75 Hz), theta (4–7.75 Hz), and alpha (8–12.75 Hz).

For regional analysis we averaged mean frontocentral, (F3, F4, F7, F8, C3, C4), temporal (T3, T4, T5, T6, T1, T2) and parietooccipital (O1, O2, P3, P4) amplitudes.

We calculated alpha peak frequencies at the occipital electrodes (O1 and O2). In addition we calculated the crude hemispheric asymmetry (absolute value of the difference between right side and left side: ABS (right-left) for all amplitude and peak frequency measures.

### Statistical methods

Categorical group differences were tested with Fisher's exact test. For the main QEEG analysis we used mean values (test one + test two + test three/3) and analyzed group differences with Mann-Whitney U tests for amplitude, frequency and asymmetry. As we used non-parametric tests, no log transformations were necessary. We performed subgroup analyses on AUDS patients using anti-epileptic treatment (compared to those who did not and the MDE group) with Kruskall-Wallis tests. Possible significant outcomes were tested with post-hoc Mann-Whitney U tests to identify the deviating group. In order to estimate possible drug or abstinence influence on the EEG, we performed an additional analysis on the third test (Possible abstinence or illegal drug effects would minimally influence the test 10 days after admittance). We studied multiple predefined hypotheses; hence we did not apply any correction for multiple significance testing. These corrections are not generally appropriate in such studies [20,21]. Two sided p- values ≤ 0.05 are reported as significant. A two-sample Student's t-test with 32 subjects has 72% power to detect an effect = 90% of group SD.

## Results

### Clinical EEG

Eight (50%) of the AUDS patients and one (6%) of the MDE patients had two or more abnormal features after three registrations (p = 0.016, Table 2). More AUDS than MDE patients had abnormal EEGs: In the first test 38% of AUDS patients and 25% of MDE patients had abnormal EEG. Epileptiform sharp activity, focal slow activity, and generalized slow activity were all found in more AUDS patients than MDE patients, but differences did not reach statistical significance.

**Table 2:** Visually interpreted (clinical) EEG results

	AUDS patients n = 16	MDE patients n = 16	p value^a^
Focal slow activity	5 (31%)	3 (19%)	0.69

General slow activity	5 (31%)	2 (13%)	0.39

Epileptiform activity	5 (31%)	2 (13%)	0.39

**0–1 abnormal feature**	**8 (50%)**	**15 (94%)**	**0.016^b^**

**≥ 2 abnormal features**	**8 (50%)**	**1 (6%)**	

Number of subjects (%) with abnormal EEG-features in at least one of three tests^a^Fisher's exact test. AUDS: Acute Unstable Depressive Syndrome. MDE: Major Depressive Episode

^b^The cutoff employed is the median number of abnormal features

### QEEG

**Figure 1:**
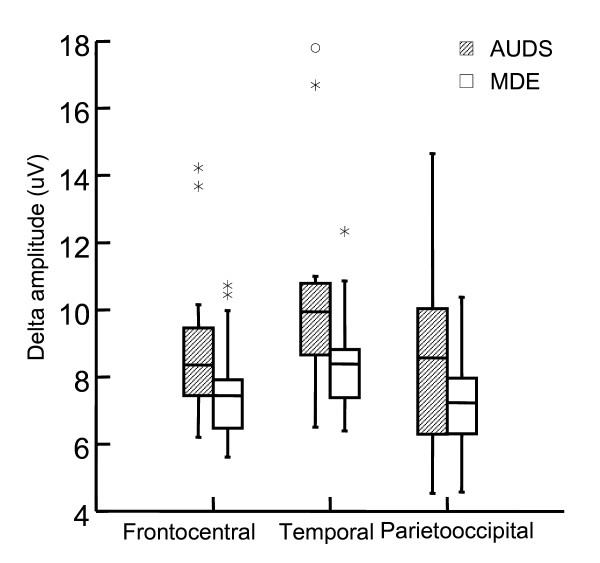
**Delta amplitude in frontocentral temporal and parietooccipital regions**. The difference is significant in the temporal region (p = 0.03), and tends to be significant in the frontocentral region (p = 0.08, Mann-Whitney U tests) (Table 3). Box-and whisker plot shows median, ± 25% percentile (box) and range. μV: micro volt, the y axis in the amplitude- (square root of power) spectrum.

**Figure 2:**
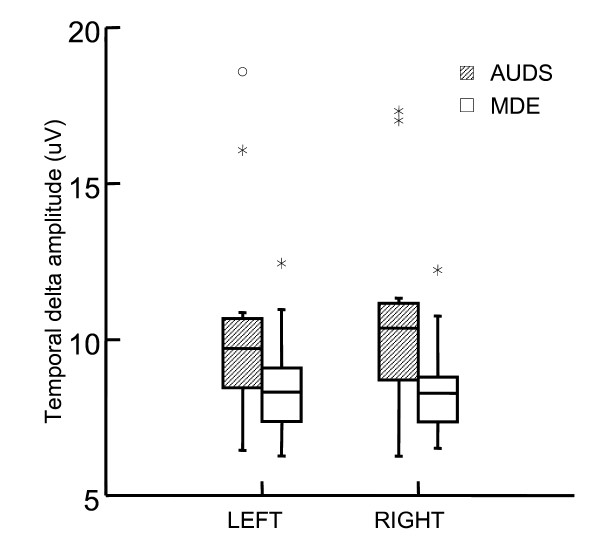
**Temporal delta amplitude on the left and the right side**. AUDS: Acute Unstable Depressive Syndrome. MDE: Major Depressive Episode. AUDS patients had significantly higher delta amplitude on the right side compared to MDE patients (p = 0.02) while a trend is observed in the left temporal region (p = 0.09; Mann-Whitney U tests). μV: micro volt, the y axis in the amplitude- (square root of power) spectrum.

**Figure 3:**
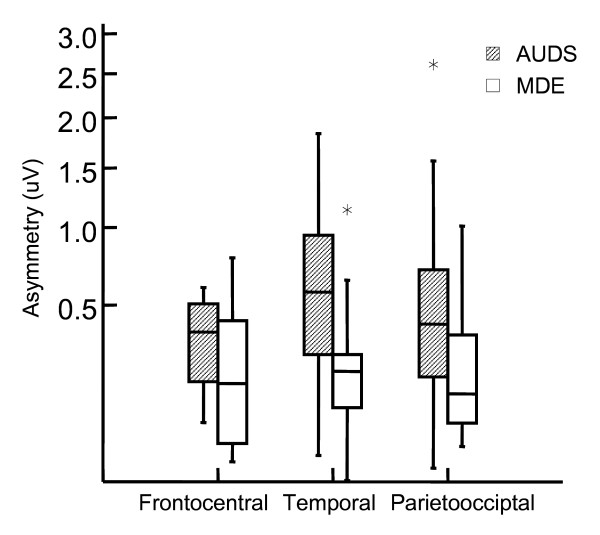
**Absolute side difference in delta amplitude in frontocentral, temporal and parietooccipital regions**. The most significant difference is observed for temporal delta asymmetry (p = 0.009), while trends are observed for parietooccipital (p = 0.05) and frontocentral delta asymmetry (p = 0.08; Mann-Whitney U tests). AUDS: Acute Unstable Depressive Syndrome. MDE: Major Depressive Episode. μV: micro volt, the y axis in the amplitude- (square root of power) spectrum.

We found significantly higher temporal delta band amplitude in AUDS patients- than in MDE patients (Table 3, Figure 1). Delta activity was most prominent in the right hemisphere (Figure 2). Delta asymmetry was larger in AUDS than MDE in all regions (Table 4, Figure 3). Twelve of 16 AUDS patients had temporal delta amplitude above 8.9 μV as compared to 3 of 16 MDE patients (sensitivity 76%, specificity 81% for this cut-off). Three MDE – and 11 AUDS patients had absolute temporal delta amplitude side difference above 0.1 μV (sensitivity 69%, specificity 81% for this cut-off). These cutoffs were selected by trial and error after inspection of the box-and-whisker plots as those that yielded the optimal separation between the two groups.

**Table 3:** Quantitative electroencephalography (QEEG) findings

	AUDS patients n = 16	MDE patients n = 16	p-value^a^
**Delta activity (μV)**			

frontocentral	8.8 (2.3)	7.6 (1.6)	0.08

temporal	**10.3 (3.1)**	**8.5 (1.6)**	**0.03**

parietooccipital	8.8 (2.9)	7.21 (1.4)	0.10

**Theta activity (μV)**			

frontocentral	7.6 (3.4)	6.9 (2.5)	0.78

temporal	9.0 (4.3)	7.8 (2.5)	0.67

parietooccipital	8.5 (4.2)	7.5 (2.8)	0.56

**Alpha activity (μV)**			

frontocentral	10.9 (4.8)	11.7 (4.6)	0.59

temporal	12.4 (5.8)	12.7 (4.8)	0.62

parietooccipital	14.9 (9.3)	15.1 (5.8)	0.42

**Alpha peak frequency (Hz)**			

occipital	9.94 (0.63)	10.24 (0.73)	0.27

occipital absolute side difference	0.86 (0.71)	0.46 (0.38)	0.13

Mean regional spectral amplitudes in μV (Standard deviation, SD) and alpha peak frequency in Hz (SD). Test 1, 2 and 3, (left and right side) values have been averaged.

^a^Mann-Whitney U test,

AUDS: Acute Unstable Depressive Syndrome. MDE: Major Depressive Episode

**Table 4:** Asymmetry in QEEG parameters

	AUDS patients n = 16	MDE patients n = 16	p-value^a^
**Frontocentral region (μV)**			

delta	0.35 (0.18)	0.25 (0.23)	0.076

theta	0.25 (0.20)	0.33 (0.27)	0.52

alfa	0.41 (0.33)	0.50 (0.51)	0.79

**Temporal region (μV)**			

**delta**	**0.66 (0.53)**	**0.27 (0.28)**	**0.009**

theta	0.53 (0.37)	0.40 (0.30)	0.12

alfa	1.18 (1.48)	0.85 (0.74)	0.85

**Parietooccipital region (μV)**			

**delta**	**0.60 (0.68)**	**0.26 (0.29)**	**0.05**

theta	0.60 (0.81)	0.49 (0.61)	0.82

alfa	1.05 (0.95)	0.96 (1.20)	0.35

Absolute difference in μV (SD) between right and left side electrode regions.

Test 1, 2 and 3 values have been averaged.

^a^Mann-Whitney U test,

AUDS: Acute Unstable Depressive Syndrome. MDE: Major Depressive Episode

Although the AUDS patients had slower occipital alpha peak frequency than the MDE patients, neither this difference nor the peak frequency asymmetry was significantly different between groups (Table 3).

### Background data

Drowsiness and AUDIT scores did not differ between groups (Table 1). Comparing band amplitudes in the third test only (in order to eliminate possible effects of drug or alcohol abstinence) did confirm our main result: increased temporal delta amplitude was found in AUDS patients compared to the MDE patients (10.3 μV vs. 8.5 μV, p = 0.04). More AUDS patients than MDE patients used anti-epileptic medication (Table 1). Some patients used more than one medicament: seven patients were on lamotrigine, one patient used valproate, and three patients used carbamazepine, while four patients and one control used clonazepam. However, subgroup analyses showed that antiepileptic drug use did not influence any of the significant results in Table 3 and 4. Indeed the AUDS subgroup not using anti-epileptic drugs did also have significantly more temporal delta activity than MDE patients (mean (SD) 9.4 (0.8) μV vs. 8.5 (1.6) μV, p = 0.04).

## Discussion

More AUDS than MDE patients had multiple abnormal features in their EEG. Other studies, suggest that individuals with EEG abnormalities within the affective disorder group may have another symptomatology and aetiology of the disorder. [12]. These patients may have an organic basis for the disease [22-25]. Our findings accordingly suggest that more AUDS than MDE patients may have an organic origin to their symptoms.

QEEG delta amplitude was significantly higher in AUDS than MDE patients. QEEG is a more sensitive method than visual EEG inspection [15]. Delta activity is a relatively unspecific finding. Slow waves between 0.5 and 4 Hz prevail naturally during sleep [26]. Pathological polymorphic delta activity can be elicited by lesions of the subcortical white matter, the thalamus and the mesencephalic reticular formation [27] most likely produced by the pyramidal neurons of the cortex [28]. In cats, localized delta activity appears in cortex overlying a circumscribed white matter lesion, but may also result from a localized thalamic lesion. Unilateral diffuse delta activity appears on the side of thalamic or hypothalamic lesions, whilst bilateral delta activity results from bilateral lesions of the midbrain tegmentum [29]. Presumably, diminished cholinergic influence from cortical projecting neurons of the basal forebrain induces the delta activity [26]. Localized lesions of the cerebral cortex do not produce delta activity on the other hand [29]. Generally, either a structural lesion, a metabolic abnormality, or lesional epileptiform activity should be considered [30].

Increased asymmetry with higher delta power on the right side was observed in the AUDS group. Hemispheric asymmetry with right sided dominance found in psychosis has been attributed to left side deficiency with the right side becoming dominant because of a lack of interhemispheric suppression [31]. Epilepsy should also be considered, as recurrent brief depressive episodes have been described both in postictal dysphoria and interictal dysphoric disorder in epilepsy [32]. A temporal epileptogenic lesion tend to be associated with polymorphic slow activity or runs of rhythmic delta or theta activity [33], but neither EEG-pattern was observed. Visual EEG did not provide evidence for significantly increased epileptiform activity. Lesional epilepsy is accordingly less likely in AUDS.

Decreased delta activity in MDE patients, as opposed to increased activity in the AUDS group, could have contributed to the observed group difference [34]. However, as more clinical EEG abnormalities were also seen in AUDS than in MDE patients, it seems likely that the group difference originates from excess delta activity in AUDS patients. Healthy controls must also be studied with a larger cohort of MDE and AUDS patients to settle this question. In addition it cannot be ruled out that a depression-related factor in the AUDS group explains or contributes to the observed EEG asymmetry [35], as elevated right side delta power has been reported in depressed patients [36]. However this is less likely, as depression mostly has been associated with increased alpha and beta power and asymmetry rather than increased slowing [37,38]. The use of other EEG-analytical methods seems to indicate a more diverse broad-band dysfunction in depressives [39]. Asymmetry was not found among unselected depressives in a recent blinded study [34].

The co-existing psychiatric symptoms in the AUDS patients could naturally contributed to the results. Coexistent anxiety may be linked with right frontal EEG dysfunction in depression [40]. John et al [31] have found power asymmetry, with more power in the right side in all or almost all bands to be a common feature of psychotic patients independent of whether the psychosis were due to schizophrenia, depression or alcohol as well as medication status. Half of the AUDS population was co-diagnosed with psychosis; hence this state may have contributed to the elevated asymmetry numbers. Although the nosological status of AUDS patients presently is not clear, our results also suggest that presence of co-morbidity should be paid even more attention to in future EEG-studies in depression.

Other explanations for EEG-slowing should also be considered. First, substance use may affect EEG. Slow EEG activity after an alcohol-related seizure has been observed [41,42], but in the present material, alcohol use was similar between groups. Moreover, EEG recorded 8–10 days after admittance, (free from any pre-hospital medication influence) confirmed our main results. Second, antiepileptic drugs, like phenytoin and carbamazepine are known to cause EEG slowing [43] or QEEG slowing [44]. We had a possible medication bias in our material caused by the use of antiepileptic drugs used as psychiatric stabilizing agents in the study group. Treatment was necessary for ethical reasons. However, the group without antiepileptic medication alone had significantly more delta activity than the controls even though our subgroup analyses were not powered to detect such differences. The results are therefore most likely not produced by the drug administration.

The limited number of patients renders our results vulnerable to type II errors. We emphasize that independent confirmation is needed. It might be argued that pre-hospital circumstances are hard to control, and it is uncertain if a high sensitivity and specificity of QEEG can be achieved in a clinical setting. However, this study aimed at investigating patients "as they are" in a realistic acute psychiatric setting because the diagnostic challenge arises here, not in a controlled environment. We have used a well-matched control group admitted under equal circumstances as these "real life patients". EEG is nevertheless useful for the diagnostic process in acute psychiatric settings [10] e.g. for evaluation of disorders like epilepsy, dementia and encephalopathy. With the development of large, commercially available QEEG databases, some QEEG techniques may grow towards clinical utility [13].

## Conclusion

Our results suggest that a group of patients with rapidly fluctuating psychiatric symptoms including depression have more abnormal EEG, compared to patients with MDE. The findings suggest more pronounced organic brain dysfunction in AUDS- than in MDE patients. Although the hypothesized association between epilepsy and AUDS was not statistically confirmed in the present study, a subgroup of AUDS patients had epileptiform activity in their EEG. The nosological status of these patients is not yet clear and a more detailed study of clinical and neuroradiological features is underway.

## Competing interests

Dr Morken received a travel grant from Astra Zeneca, but none of the authors have any financial interests or other potential conflicts of interest.

## Authors' contributions

AEV, GB, GM and OML conceived, designed and coordinated the study, examined and included the patients and helped to draft the manuscript. TS planned and supervised the EEG procedures and interpreted the EEGs. BMN did the MATLAB programming. MHB and TS extracted the data, performed the statistical analyses and drafted the manuscript. All authors read and approved of the final manuscript.

## Pre-publication history

The pre-publication history for this paper can be accessed here:


